# A combined score based on anterior chest wall CT attenuation to aid recognition of SAPHO syndrome: a training-validation study

**DOI:** 10.3389/fmed.2026.1910778

**Published:** 2026-09-10

**Authors:** Fengyu Liu, Liujie Zheng, Sujuan Xu, Lin Liu, Tao Wang, Haoyuan Fu, Guoqiang Li, Erdong Zhang, Ling Wang, Feng Luan, Zhiyong Hou

**Affiliations:** 1 Postdoctoral Mobile Station of Hebei Medical University Third Hospital, Shijiazhuang, Hebei, China; 2 Department of Orthopedic Surgery, Hebei Medical University Third Hospital, Shijiazhuang, Hebei, China; 3 Department of Nephrology, Hebei Medical University Third Hospital, Shijiazhuang, Hebei, China; 4 Orthopaedic Research Institute of Hebei Province, Shijiazhuang, Hebei, China; 5 Department of Lower Limb Trauma, Beijing Jishuitan Hospital, Guizhou Hospital, Guiyang, Guizhou, China; 6 Department of Otorhinolaryngology, Hebei Medical University Third Hospital, Shijiazhuang, Hebei, China; 7 Key Laboratory of Biomechanics of Hebei Province, Shijiazhuang, Hebei, China; 8 NHC Key Laboratory of Intelligent Orthopaedic Equipment, Shijiazhuang, Hebei, China; 9 Engineering Research Center of Orthopedic Minimally Invasive Intelligent Equipment, China Ministry of Education, Shijiazhuang, China; 10 Key Laboratory of Precise Assessment, Diagnosis, and Treatment of Soft Tissue Injury of Hebei Province, Shijiazhuang, China

**Keywords:** anterior chest wall, combined score, CT attenuation, quantitative CT, SAPHO syndrome

## Abstract

**Objective:**

To investigate whether quantitative anterior chest wall CT attenuation can aid recognition of SAPHO syndrome, to develop a simple combined score, and to evaluate its discriminatory performance in a validation set.

**Methods:**

We retrospectively enrolled 260 patients with SAPHO syndrome and 260 age- and sex-matched healthy controls. CT attenuation values were measured at five sites: the left and right clavicular heads, the left and right upper manubrium, and the sternal angle, and were normalized to the CT attenuation of an internal reference vertebra. Using stratified random sampling (7:3), the total sample was divided into a training set (364 cases: 182 SAPHO and 182 controls) and a validation set (156 cases: 78 SAPHO and 78 controls). In the training set, ROC analysis was used to compare individual ratios and additive combinations, establish the combined score, and determine the optimal cutoff. The fixed cutoff was then applied to the validation set. In addition, a subgroup analysis was performed in SAPHO patients without obvious anterior chest wall CT morphological changes.

**Results:**

In the training set, the combined score achieved an AUC of 0.980 (95% CI: 0.969–0.991). At the optimal cutoff of >5.412, sensitivity and specificity were both 93.41%. In the validation set, using the same cutoff, the AUC was 0.976 (95% CI: 0.958–0.993), with a sensitivity of 92.3% and a specificity of 84.6%. Among 96 SAPHO patients without obvious anterior chest wall CT morphological changes, 81 (84.4%) had combined scores above the fixed cutoff; the AUC for distinguishing this subgroup from healthy controls was 0.956 (95% CI: 0.935–0.974).

**Conclusion:**

The combined score based on multi-site anterior chest wall CT attenuation demonstrated high discriminatory ability between SAPHO patients and healthy controls and showed consistent performance in the internal validation set. In patients without obvious anterior chest wall CT morphological changes, the score provided additional quantitative imaging information and may serve as an objective complement to conventional morphological assessment.

## Introduction

SAPHO syndrome (synovitis, acne, pustulosis, hyperostosis, osteitis) is a rare chronic inflammatory disorder. One of its typical manifestations is sterile osteitis and hyperostosis of the anterior chest wall, particularly the sternocostoclavicular region (1). Owing to its diverse clinical presentations and the lack of specific biomarkers, SAPHO syndrome is frequently underdiagnosed or misdiagnosed, resulting in delayed treatment (2, 3). Imaging examinations, especially computed tomography (CT), can clearly demonstrate hyperostosis, sclerosis, and bony bridging of the sternum, clavicles, and first ribs, and thus play an important role in diagnosis (4).

However, routine CT evaluation relies primarily on morphological features such as sclerosis, hyperostosis, and bony bridging, and subtle or atypical osseous changes may not be readily identified by visual assessment alone (5, 6). In recent years, CT attenuation-based bone assessment has been widely used for opportunistic evaluation of osteoporosis (7, 8), indicating that routine CT images contain quantitative bone information beyond conventional morphology. The anterior chest wall is one of the most commonly affected regions in SAPHO syndrome, and cancellous bone attenuation may reflect local osteitis and abnormal bone remodeling (9–12). Therefore, quantitative analysis of existing chest CT images may provide objective imaging information complementary to conventional morphology, particularly when anterior chest wall changes are not obvious.

Based on these considerations, we hypothesized that relative CT attenuation values of the anterior chest wall differ between patients with SAPHO syndrome and healthy individuals, and that a multi-site combined score could further improve discriminatory performance. To our knowledge, quantitative CT attenuation of the anterior chest wall has not been systematically evaluated for this purpose. Therefore, the aims of this study were: (1) to compare CT attenuation ratios, normalized to an internal reference vertebra, at five anterior chest wall sites (left and right clavicular heads, left and right upper manubrium, and sternal angle) between SAPHO patients and healthy controls; (2) to identify the best-performing additive combination and define it as the combined score; and (3) to evaluate the discriminatory performance of the combined score using a training-validation design. In addition, we assessed the performance of the combined score in SAPHO patients without obvious anterior chest wall CT morphological changes.

## Materials and methods

### Study population

**Figure 1:**
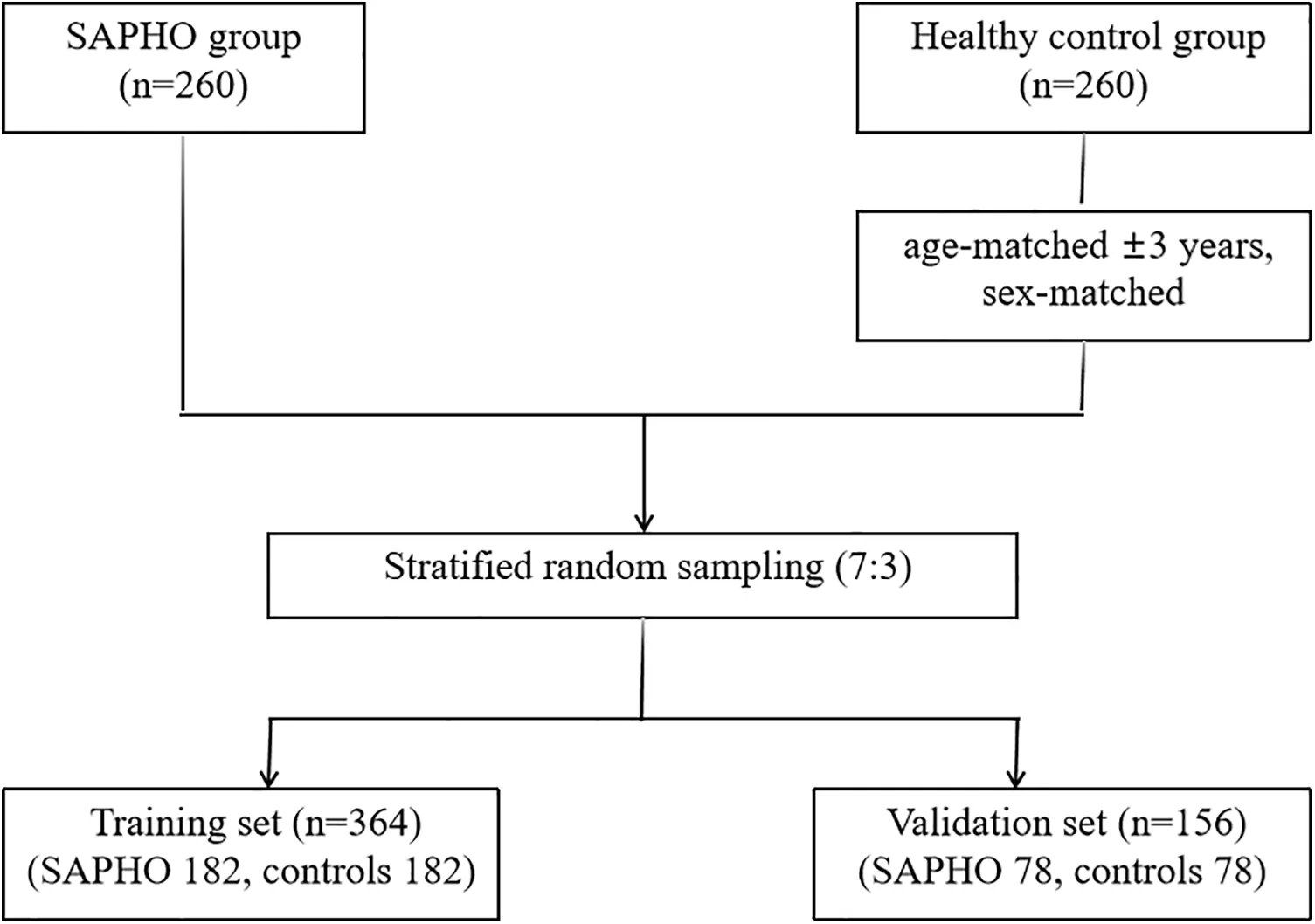
Flowchart of study population selection and data split. SAPHO group (*n* = 260) and healthy control group (*n* = 260) were matched by age (±3 years) and sex. Using stratified random sampling at a ratio of 7:3, the total sample was divided into a training set (*n* = 364: 182 SAPHO, 182 controls) and a validation set (*n* = 156: 78 SAPHO, 78 controls).

This study was approved by the Institutional Ethics Committee. Written informed consent was waived due to the retrospective nature of the study. A total of 260 patients with SAPHO syndrome diagnosed at the SAPHO Center of our hospital between January 2023 and May 2026 were consecutively enrolled. SAPHO syndrome was diagnosed according to the criteria proposed by Kahn et al. (13–16), integrating clinical and imaging findings. All included patients had an established clinical diagnosis, underwent whole-body bone scintigraphy, and showed abnormal osseous uptake. Quantitative anterior chest wall CT attenuation measurements and combined-score calculations were performed retrospectively after the clinical diagnosis had been established; these quantitative CT parameters were not used in the diagnostic process. In addition, 260 age- and sex-matched individuals (age ± 3 years) who underwent routine chest CT at the health examination center of our hospital were included as healthy controls. Controls had no history of osteoarticular diseases, autoimmune disorders, or chronic inflammatory conditions (Figure 1). Exclusion criteria were: (1) history of sternal or clavicular trauma or surgery; (2) spinal or thoracic deformities that could affect CT measurements; and (3) bone metastases from malignancy.

### CT acquisition and measurement

All subjects underwent non-contrast chest CT using a 64-slice helical CT scanner (Siemens Definition AS, Germany). The scanning parameters were as follows: tube voltage 120 kV, tube current automatically modulated (reference 250 mA), slice thickness 1.25 mm, and pitch 1.2. The scan range extended from the thoracic inlet to the diaphragm. Images were transferred to a PACS for storage and analysis. Quantitative CT measurements in all 520 participants were performed on the clinically reconstructed B70f series. No additional image reconstruction or post-processing was performed specifically for this study.

### Measurement protocol

**Figure 2:**
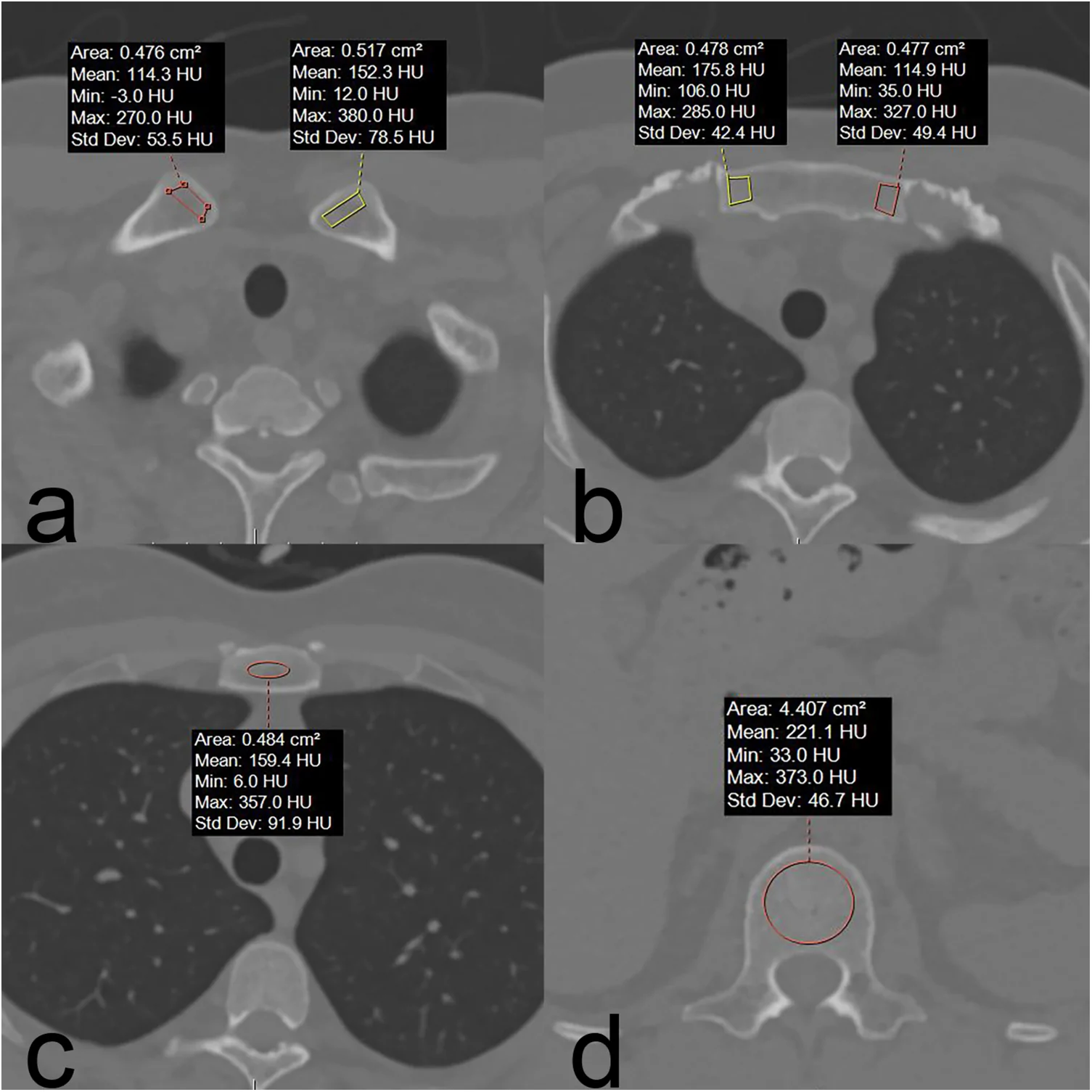
Representative measurement of CT attenuation values at the five anterior chest wall sites and the reference vertebra. Regions of interest (ROIs) were placed on axial CT images at the following sites: **(a)** left and right clavicular heads; **(b)** left and right upper manubrium at the level of the first sternocostal joint; **(c)** sternal angle; and **(d)** reference vertebra. L1 was preferred as the reference vertebra; when L1 was unsuitable because of an abnormality affecting cancellous bone attenuation, a structurally normal adjacent T12 or L2 vertebra was used. Quantitative measurements were performed on the B70f reconstruction series. The images are displayed using a wide bone-window setting (window width 4000 HU; window level 200 HU). The same measurement protocol was applied to all participants.

All routine measurements were performed by a single trained researcher who was blinded to the patients’ group status (SAPHO vs. control). Reference vertebra: The L1 vertebral body was preferred as the internal reference. The L1 vertebral body was identified on the CT scout/localizer image. The corresponding axial image at the mid-vertebral level was selected, and an elliptical ROI was placed within the cancellous bone, as large as possible while avoiding cortical bone, basivertebral veins, and focal osseous abnormalities. L1 was considered unsuitable when abnormalities likely to affect cancellous bone attenuation were present, including vertebral fracture, hemangioma, bone island, marked focal sclerosis, severe degenerative change, or another definite focal osseous lesion; in such cases, a structurally normal adjacent T12 or L2 vertebra was used. Clavicular heads: The medial-inferior aspect of each clavicular head at the sternoclavicular joint level was identified. On axial images, an ROI of approximately 0.5 cm^2^ was placed at the center of the cancellous bone of each clavicle. Upper manubrium: The upper manubrium was identified at the level of the first sternocostal joint. On axial images, separate ROIs of approximately 0.5 cm^2^ were placed in the left and right cancellous bone regions of the upper manubrium. Sternal angle: The level of the sternal angle (the junction of the manubrium and sternal body) was identified. On axial images, an ROI of approximately 0.5 cm^2^ was placed at the center of the cancellous bone of the sternum (Figure 2).

For each site, CT attenuation was measured at three different slices (1-mm intervals), and the average value was taken as the final CT attenuation for that site. To assess measurement reliability, thirty cases (15 SAPHO, 15 controls) were randomly selected from the study population. These cases were independently measured by two observers. Additionally, the same observer remeasured these cases after an interval of two weeks. The intraclass correlation coefficient (ICC) was calculated to evaluate inter-observer and intra-observer reliability.

### Data correction and combined score

To reduce inter-individual differences in baseline bone attenuation and body habitus, the CT attenuation value of each anterior chest wall site was divided by the CT attenuation value of the reference vertebra in the same participant to obtain a relative ratio. The five relative ratios were designated as follows: LC (left clavicular head/reference vertebra), RC (right clavicular head/reference vertebra), LM (left upper manubrium/reference vertebra), RM (right upper manubrium/reference vertebra), and SA (sternal angle/reference vertebra).

In the training set, simple arithmetic sums of different numbers of these five ratios were calculated, including sums of two, three, four, and all five ratios. The discriminatory performance of each combination was evaluated by comparing their AUCs, and the combination with the highest AUC was subsequently defined as the combined score.

### Training and validation set split

Using stratified random sampling, the SAPHO group and the control group were each randomly allocated to the training set and validation set at a ratio of 7:3. The final training set consisted of 364 cases (182 SAPHO and 182 controls), and the validation set consisted of 156 cases (78 SAPHO and 78 controls).

### Anterior chest wall CT morphology subgroup analysis

To evaluate the performance of the combined score in SAPHO patients without obvious morphological changes on routine CT, two radiologists independently reviewed the entire anterior chest wall in all 260 SAPHO patients, including the sternum, bilateral sternoclavicular joints, and clavicular heads, while blinded to CT attenuation values and combined-score results. Based on an overall assessment of typical SAPHO-related CT findings, including sclerosis, hyperostosis, bony bridging, and other definite structural abnormalities, patients were categorized as having obvious or non-obvious anterior chest wall morphological changes. Cases with concordant assessments were assigned directly; disagreements or uncertain cases were jointly reviewed until consensus was reached. This subgroup analysis was not used in construction of the combined score or determination of its cutoff; the cutoff derived from the training set was applied without re-estimation.

### Statistical analysis

Data analysis was performed using R version 4.5.1. Continuous variables were expressed as mean ± standard deviation or median (interquartile range), as appropriate, and comparisons between groups were made using the independent-samples t-test (for normally distributed data) or the Mann–Whitney U test (for non-normally distributed data). Categorical variables were presented as frequencies (percentages) and compared using the *χ*^2^ test. In the training set, the area under the receiver operating characteristic (ROC) curve (AUC) and 95% confidence interval were calculated for each individual ratio and for different additive combinations. The optimal cutoff was determined using the Youden index. The cutoff derived from the training set was applied to the validation set without re-estimation, and the corresponding sensitivity and specificity were calculated. The DeLong test was used to compare AUCs. For the morphology subgroup analysis, combined-score distributions among the three groups were compared using the Kruskal–Wallis test, followed by pairwise Mann–Whitney U tests with Holm correction. The predefined cutoff derived from the training set was directly applied to both SAPHO morphological subgroups, and the proportions exceeding the cutoff were compared using the *χ*2 test. ROC analyses were further performed to assess the discriminatory performance of the combined score for each SAPHO morphological subgroup versus healthy controls. All tests were two-sided, and a *p*-value < 0.05 was considered statistically significant.

## Results

### Baseline characteristics and measurement reliability

A total of 260 patients with SAPHO syndrome (182 females, 70.0%; mean age 42.3 ± 9.8 years) and 260 age- and sex-matched healthy controls (182 females, 70.0%; mean age 42.9 ± 9.9 years) were enrolled. There were no statistically significant differences between the two groups in age, sex, or body mass index (BMI) (*p* > 0.05). The median disease duration in SAPHO patients was 24 months (IQR: 7–60 months), and anterior chest wall pain was reported in 206 (79%) patients. Baseline characteristics are summarized in Table 1.

**Table 1:** Baseline characteristics of the study population (full dataset).

Variable	SAPHO (*n* = 260)	Control (*n* = 260)	*P*
Age (years, mean ± SD)	42.3 ± 9.8	42.9 ± 9.9	0.434
Female sex, *n* (%)	182 (70%)	182 (70%)	1
BMI (kg/m^2^, mean ± SD)	24.6 ± 3.7	24.4 ± 3.5	0.574
Disease duration (months, median [IQR])	24 [7−60]		
Anterior chest wall pain, *n* (%)	206 (79%)		

Thirty cases were randomly selected for repeated measurement analysis. The intra-observer ICC for CT attenuation values of each site ranged from 0.88 to 0.97, and the inter-observer ICC ranged from 0.86 to 0.94, indicating good reliability of the averaged values from three measurements.

### Comparison of relative CT attenuation ratios between groups in the training set

In the training set, all five relative CT attenuation ratios were significantly higher in SAPHO patients than in healthy controls (all *p* < 0.001). The detailed comparisons are presented in Table 2.

**Table 2:** Comparison of relative CT attenuation ratios between SAPHO patients and healthy controls in the training set.

Ratio	SAPHO (*n* = 182)	Control (*n* = 182)	*P*
Left clavicular head/reference vertebra (median [IQR])	1.48 [1.24–1.85]	0.84 [0.61–1.00]	< 0.001
Right clavicular head/reference vertebra (median [IQR])	1.42 [1.19–1.77]	0.80 [0.58–1.03]	< 0.001
Left upper manubrium/reference vertebra (median [IQR])	1.58 [1.07–2.56]	0.78 [0.59–0.95]	< 0.001
Right upper manubrium/reference vertebra (median [IQR])	1.52 [1.14–2.30]	0.82 [0.68–0.98]	< 0.001
Sternal angle/reference vertebra (median [IQR])	1.25 [0.97–1.82]	0.67 [0.54–0.79]	< 0.001

### Discriminatory performance of individual ratios in the training set

**Figure 3:**
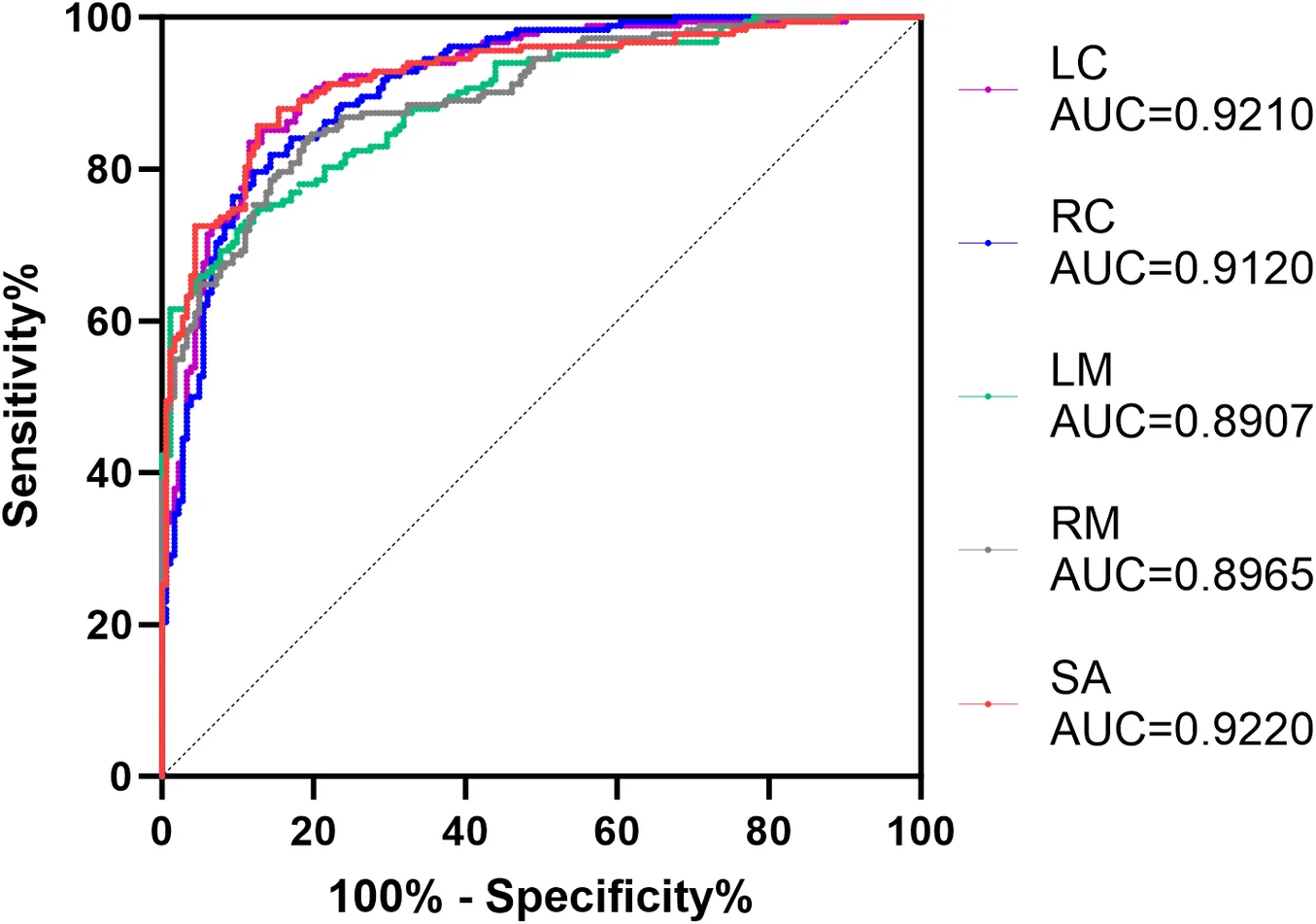
ROC curves of individual CT attenuation ratios in the training set. ROC curves and corresponding areas under the curve (AUC) for the five individual ratios: LC (left clavicular head/reference vertebra), RC (right clavicular head/reference vertebra), LM (left upper manubrium/reference vertebra), RM (right upper manubrium/reference vertebra), and SA (sternal angle/reference vertebra). The sternal angle/reference vertebra ratio achieved the highest AUC among individual ratios (0.9220).

In the training set (364 cases), the ROC analysis results for each individual ratio are shown in Figure 3. Among these, the sternal angle/reference vertebra ratio yielded the highest AUC of 0.9220 (95% CI: 0.8940–0.9499), with an optimal cutoff of >0.8972, a sensitivity of 85.71%, and a specificity of 87.36%. The AUCs of the remaining ratios ranged from 0.8907 to 0.9210 (Table 3).

**Table 3:** ROC analysis results of CT attenuation ratios of each region in the training set.

Ratio	AUC (95% CI)	Optimal threshold	Sensitivity (%)	Specificity (%)
LC	0.9210 (0.8936–0.9484)	>1.167	83.52	88.46
RC	0.9120 (0.8834–0.9406)	>1.136	79.67	87.91
LM	0.8907 (0.8585–0.9230)	>1.125	71.98	90.11
RM	0.8965 (0.8652–0.9277)	>1.019	84.62	80.22
SA	0.9220 (0.8940–0.9499)	>0.8972	85.71	87.36
LC + RC + LM + RM + SA	0.9799 (0.9691–0.9907)	>5.412	93.41	93.41
LC + RC + LM + RM	0.9692 (0.9544–0.9840)	>4.492	91.76	91.76
LC + RC + SA	0.9692 (0.9553–0.9831)	>3.057	91.21	89.01
LM + RM + SA	0.9594 (0.9427–0.9762)	>2.886	92.31	84.62
LC + RC	0.9361 (0.9122–0.9599)	>2.091	89.56	84.62
LM + RM	0.9246 (0.8991–0.9500)	>1.984	89.01	80.22

LC, left clavicular head/reference vertebra; RC, right clavicular head/reference vertebra; LM, left upper manubrium/reference vertebra; RM, right upper manubrium/reference vertebra; SA, sternal angle/reference vertebra.

### Construction and performance of the combined score in the training set

**Figure 4:**
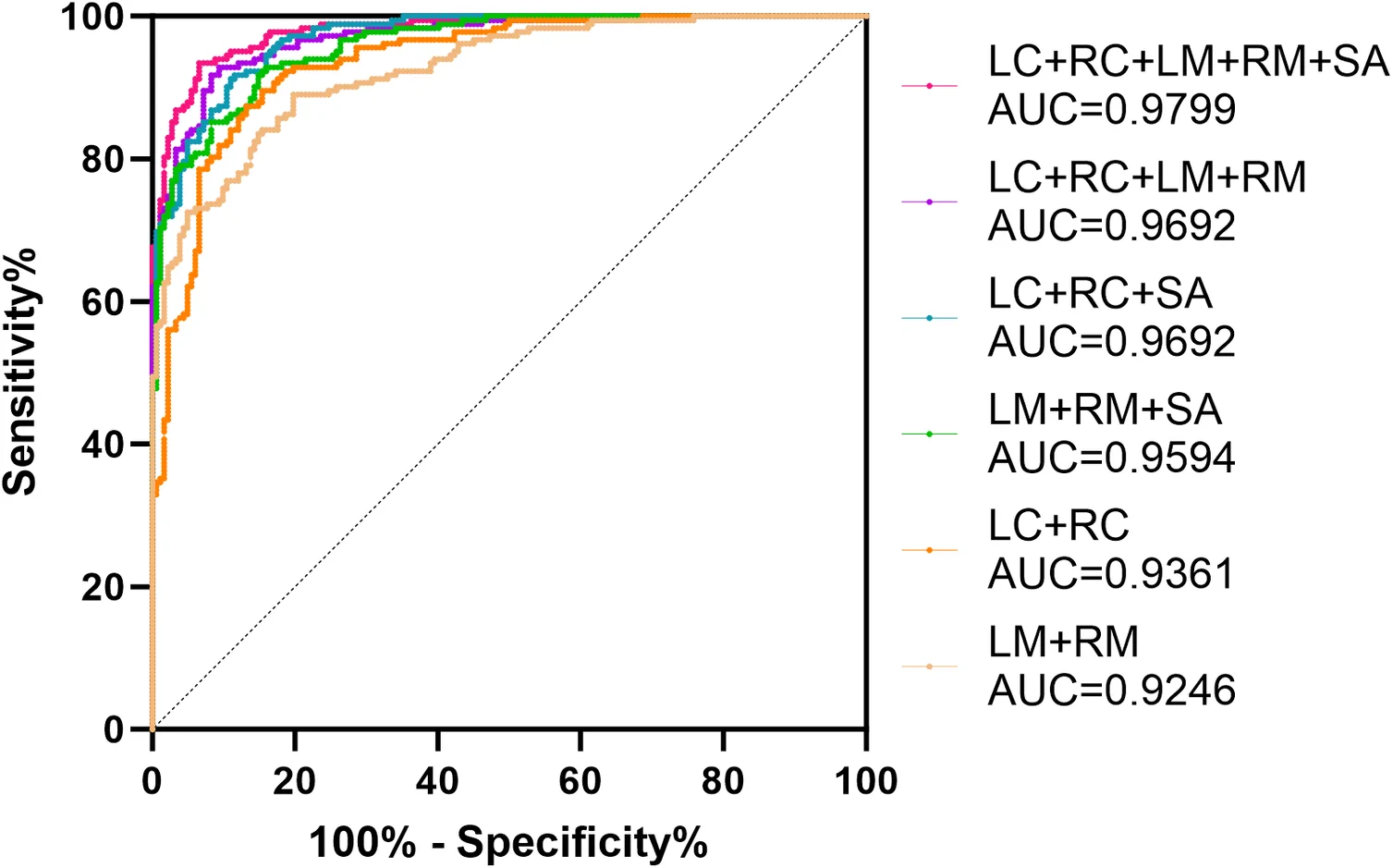
ROC curves of different additive combinations of the five ratios in the training set. Various arithmetic sums of the five ratios were evaluated. The sum of all five ratios (LC + RC + LM + RM + SA) showed the best discriminatory performance, with an AUC of 0.9799. Other combinations, including sums of four, three, and two ratios, are also presented. This five-ratio sum was defined as the combined score.

In the training set, simple arithmetic sums of different numbers of site-specific ratios were calculated, and the area under the ROC curve (AUC) of each combination was compared (Figure 4). The sum of all five ratios (LC + RC + LM + RM + SA) exhibited the best discriminatory performance, with an AUC of 0.9799 (95% CI: 0.9691–0.9907). The DeLong test showed that this AUC was significantly higher than that of the best individual ratio (sternal angle/reference vertebra, AUC = 0.9220) (*p* < 0.001). The optimal cutoff determined by the Youden index was >5.412, yielding a sensitivity of 93.41% and a specificity of 93.41%. The AUCs and performance of the other combinations (e.g., sums of four or three sites) are also presented in Table 3. Based on these findings, the simple arithmetic sum of the five ratios was defined as the combined score and used for subsequent validation.

### Discriminatory performance of the combined score in the validation set

**Figure 5:**
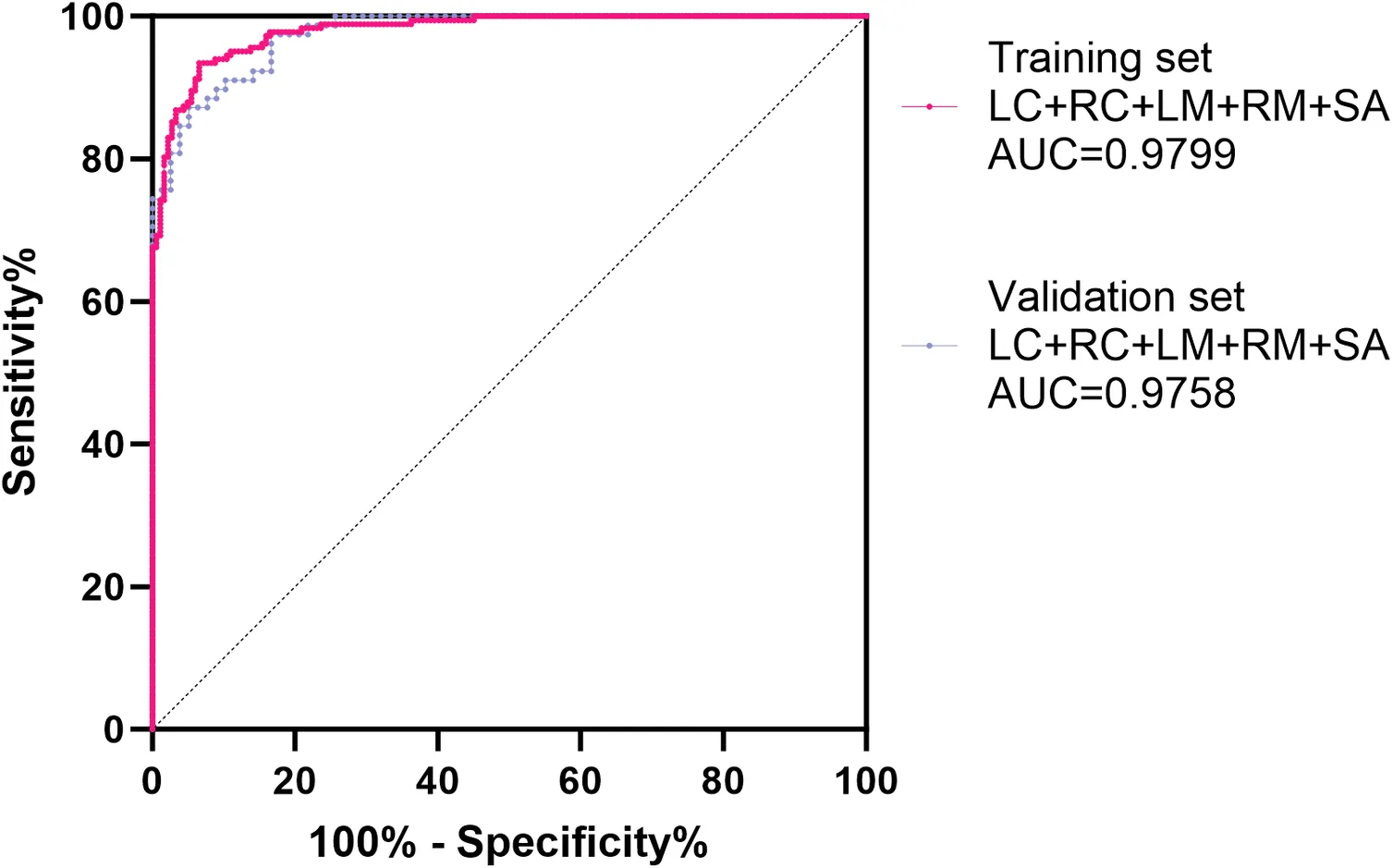
ROC curves of the combined score in the training set and validation set. The combined score (sum of LC + RC + LM + RM + SA) achieved an AUC of 0.9799 in the training set and 0.9758 in the validation set. The two AUCs did not differ significantly (*p* = 0.6955), indicating consistent discriminatory performance in the held-out internal validation set.

In the validation set (156 cases), the AUC of the combined score for distinguishing SAPHO patients from healthy controls was 0.9758 (95% CI: 0.9583–0.9934) (Figure 5). Using the fixed cutoff (>5.412) derived from the training set, the sensitivity was 92.3% and the specificity was 84.6%. Compared with the training set, sensitivity remained similar (93.4% vs. 92.3%), whereas specificity showed a modest decrease (93.4% vs. 84.6%). The DeLong test revealed no statistically significant difference between the AUCs of the training set (0.9799) and the validation set (0.9758) (*p* = 0.6955), indicating consistent discriminatory performance in the held-out internal validation set.

### Performance of the combined score according to anterior chest wall CT morphology

Among the 260 SAPHO patients, 164 had obvious anterior chest wall CT morphological abnormalities and 96 had no obvious morphological abnormalities (Table 4). Combined scores differed significantly among healthy controls and the two morphological subgroups (Kruskal–Wallis test, *P* < 0.001), with all pairwise comparisons remaining significant after Holm correction (all *P* < 0.001). Using the fixed cutoff of 5.412 derived from the training set, 161 of 164 patients (98.2%) with obvious morphological abnormalities and 81 of 96 patients (84.4%) without obvious morphological abnormalities had scores above the cutoff; the difference between the two SAPHO subgroups was significant (*P* < 0.001). In patients without obvious morphological abnormalities, the combined score yielded an AUC of 0.956 (95% CI: 0.935–0.974) for distinguishing SAPHO patients from healthy controls.

**Table 4:** Combined CT attenuation score according to anterior chest wall morphological findings.

Variable	Healthy controls	SAPHO without obvious morphological abnormalities	SAPHO with obvious morphological abnormalities
No. of subjects	260	96	164
Combined score, median (IQR)	3.98 (3.21–4.62)	6.56 (5.77–7.74)	9.20 (7.74–10.97)
Score >5.412, *n* (%)	24 (9.2%)	81 (84.4%)	161 (98.2%)
AUC vs. healthy controls (95% CI)	—	0.956 (0.935–0.974)	0.991 (0.983–0.996)

The cutoff (>5.412) was predefined in the training set and applied to both morphological subgroups without re-estimation. AUC, area under the receiver operating characteristic curve; IQR, interquartile range.

## Discussion

This study developed a simple combined score based on relative CT attenuation at five anterior chest wall sites and evaluated its ability to distinguish SAPHO patients from healthy controls using a training-validation design. Relative CT attenuation values at the bilateral clavicular heads, bilateral upper manubrium, and sternal angle were significantly higher in SAPHO patients. The five-site combined score achieved an AUC of 0.9799 in the training set and showed similar discriminatory performance in the internal validation set. Importantly, among 96 SAPHO patients without obvious anterior chest wall CT morphological changes, 81 (84.4%) still had combined scores above the fixed cutoff, and the AUC for distinguishing this subgroup from healthy controls was 0.956. These findings indicate that quantitative anterior chest wall CT attenuation can provide objective imaging information beyond conventional morphology and may improve recognition of SAPHO-related anterior chest wall bone abnormalities.

Chest CT provides a practical platform for this quantitative assessment because patients with anterior chest wall symptoms may undergo chest CT during routine clinical work-up to exclude cardiopulmonary disease, chest wall tumors, or other conditions (3). Existing chest CT images therefore provide a readily available dataset without additional scanning or radiation exposure. CT also clearly depicts the sternum, clavicles, and sternocostal structures without the superimposition inherent to plain radiography (17), while Hounsfield units provide continuous quantitative information in addition to morphology. Thus, the proposed score is intended not to replace the clinical diagnosis of SAPHO syndrome, but to extract objective information from already available CT images that may support recognition of SAPHO-related anterior chest wall abnormalities and prompt further clinical evaluation.

The bilateral clavicular heads, bilateral upper manubrium, and sternal angle were selected because the anterior chest wall is one of the most frequently involved regions in SAPHO syndrome. Previous studies have reported anterior chest wall involvement in approximately 65%–95% of adult patients (16, 18), and 79% of patients in our cohort reported anterior chest wall pain. Yu et al. (19) identified several common patterns of anterior chest wall involvement on MRI, including the first-rib region, sternoclavicular region, sternal angle, and second-to-sixth sternocostal joint regions. The five measurement sites used in the present study cover several of these characteristic locations and may therefore provide a more comprehensive representation of anterior chest wall bone abnormalities than a single-site measurement.

In the present study, anterior chest wall CT attenuation values were normalized to an internal reference vertebra. CT attenuation is influenced by individual bone mass, body habitus, and technical factors, and previous quantitative CT studies have shown that vertebral attenuation measurements, particularly at L1, can provide useful quantitative information on bone status (20, 21). L1 was preferred because it is readily identified on routine chest CT. Previous studies have shown that spinal involvement in SAPHO syndrome occurs more frequently in the thoracic and lumbar spine, although L1 is not considered a particularly predominant site of involvement (22). When L1 contained abnormalities likely to influence cancellous bone attenuation, a structurally normal adjacent T12 or L2 vertebra was used instead. Using relative ratios rather than absolute Hounsfield units may reduce inter-individual variation and improve comparability. In addition, all quantitative measurements in this study were performed on the same B70f reconstruction series, supporting internal consistency. The stability of this normalization approach across scanners and reconstruction conditions remains to be assessed in future multicenter studies (23).

Previous CT studies of SAPHO syndrome have focused mainly on morphological findings such as sclerosis, hyperostosis, and bony bridging (24–26). These findings are valuable in typical cases, but when structural changes are not obvious, visual assessment may provide limited information. CT attenuation offers a continuous quantitative measure of bone density. In the present study, 96 SAPHO patients were categorized by radiologists as having no obvious anterior chest wall CT morphological changes; nevertheless, 81 (84.4%) had combined scores above the fixed cutoff of 5.412, and the AUC for distinguishing this subgroup from healthy controls remained 0.956. These results indicate that quantitative CT does not merely reproduce visually obvious sclerosis or hyperostosis, but can provide complementary information when conventional morphological findings are inconspicuous.

SAPHO-related osteitis, abnormal bone remodeling, and new bone formation provide a plausible pathological basis for increased CT attenuation (14, 18, 27, 28). In our data, the combined score was highest in patients with obvious morphological abnormalities, intermediate in patients without obvious morphological changes, and lowest in healthy controls. This clear gradient is consistent with differing degrees of anterior chest wall bone alteration and suggests that quantitative attenuation may capture abnormalities that are not fully expressed by categorical visual assessment. Because the present study is cross-sectional, however, whether these group differences represent a temporal sequence of disease evolution requires longitudinal imaging studies.

Compared with the best individual ratio (sternal angle/reference vertebra, AUC = 0.9220), the five-site combined score increased the training-set AUC to 0.9799. This improvement is consistent with the multi-site pattern of anterior chest wall involvement in SAPHO syndrome. A single-site measurement may be influenced by local anatomical variation, degeneration, bone islands, or ROI placement, whereas combining multiple anatomically relevant sites may provide a more comprehensive representation of anterior chest wall bone abnormalities. The score is also simple to calculate, requiring only direct addition of five relative ratios and no complex modeling or specialized software.

Clinically, the combined score may be most useful as an objective clue on routine chest CT in patients with unexplained anterior chest wall pain, sternoclavicular symptoms, or other features raising suspicion of SAPHO syndrome. An elevated score, particularly when conventional CT morphology is inconspicuous, may increase recognition of possible SAPHO-related bone involvement and support further integrated clinical assessment. This approach uses information already contained in routine chest CT and positions quantitative attenuation as a complement to, rather than a replacement for, conventional imaging interpretation and clinical diagnosis.

This study has several strengths. First, it included 260 SAPHO patients and 260 age- and sex-matched healthy controls, representing a relatively large cohort for a rare disease. Second, the combined score and cutoff were established in the training set and then applied without re-estimation to a held-out validation set, reducing the risk of overfitting. Third, the score is simple, and CT attenuation measurements showed good intra-observer and inter-observer reliability. Finally, the additional morphology subgroup analysis demonstrated that the quantitative score can provide useful information even when anterior chest wall CT morphological changes are not obvious.

Several limitations should be acknowledged. First, this was a single-center retrospective case-control study using healthy controls; therefore, the observed discrimination primarily reflects differences between established SAPHO cases and healthy individuals and may overestimate performance in real-world differential diagnosis. Future studies should include clinically relevant disease controls, such as infectious, degenerative, inflammatory, and neoplastic anterior chest wall disorders. Second, the training and validation sets were derived from the same center, and multicenter external validation is needed to assess performance across different scanners and acquisition conditions. Third, because this study was cross-sectional, the relationship between the combined score and longitudinal disease progression or treatment-related change could not be evaluated.

## Conclusion

The combined score based on multi-site anterior chest wall CT attenuation demonstrated high discriminatory ability between SAPHO patients and healthy controls and showed consistent performance in the held-out internal validation set. In SAPHO patients without obvious anterior chest wall CT morphological changes, the score provided additional quantitative imaging information and may serve as an objective complement to conventional morphological assessment, supporting further clinical evaluation of SAPHO-related bone abnormalities.

## Data Availability

The original contributions presented in the study are included in the article/Supplementary Material, further inquiries can be directed to the corresponding author.
